# Novel feeder-cell-free 3C system promotes the transition from primed to formative pluripotency, self-renewal, and germline differentiation in rabbit embryonic stem cells *in vitro*

**DOI:** 10.3389/fcell.2026.1841436

**Published:** 2026-06-08

**Authors:** Xiao Chen, Zhihui Liu, Qing Li, Rahman Ullah, Yuan Gao, Xiyue Wang, Lanjun Liu, Fuliang Du

**Affiliations:** 1 Jiangsu Key Laboratory for Molecular and Medical Biotechnology, College of Life Sciences, Nanjing Normal University, Nanjing, China; 2 Hebei Technology Innovation Center of Cattle and Sheep Embryo, College of Animal Science and Technology, Hebei Agricultural University, Baoding, China; 3 Children’s Research Institute, University of Texas Southwestern Medical Center, Dallas, TX, United States; 4 Chengdu Institute of Biological Products Co., Ltd., Chengdu, China

**Keywords:** embryonic stem cell, feeder-cell-free culture, *in vitro* differentiation, PGCLCs, small molecule

## Abstract

**Introduction:**

Most rabbit embryonic stem (rbES) cell lines established in embryonic fibroblast feeder layer systems predominantly exhibit pluripotency features restricted to a “primed” state, which hinders their directed differentiation into reproductive germ cells and limits their capacity for germline transmission.

**Methods:**

We developed a novel 3C system containing IWP2, DZNep, and TSA in iFLY medium supplemented with basic fibroblast growth factor, human leukemia inhibitory factor, and Noggin. Pluripotency maintenance and germline differentiation were validated by gene expression analysis and induction with BMP4, stem cell factor, and epidermal growth factor.

**Results:**

The 3C system efficiently maintained the pluripotency of rbES cells with stable expression of core pluripotent genes. These cells showed upregulated expression of partial naïve markers and expressed formative pluripotent markers. Moreover, 3C cultured rbES cells were successfully differentiated into primordial germ cell like cells (PGCLCs).

**Discussion:**

The 3C culture system confers rbES cells with naïve pluripotency and self renewal ability, as well as strong potential for directed germ cell differentiation. This system provides a reliable stem cell platform for genetic modification and breeding in livestock.

## 1 Introduction

Embryonic stem (ES) cells are pluripotent cell populations produced by *in vitro* capture and culture of inner cell mass cells from an early blastocyst (Evans and Kaufman, 1981). Mouse embryonic stem (mES) cells produced in a 2i/leukemia inhibitory factor (LIF) culture system display the characteristics of pre-implantation epiblasts and exhibit a naïve pluripotent state (Ying et al., 2008). Naïve-state mES cells possess the broadest developmental potential and are capable of differentiating into all embryonic cell types, including reproductive germ cells (Kalkan et al., 2014). After embryo implantation, the expression of naïve pluripotency-associated transcription factors such as *Zfp42* and *Klf4* is gradually downregulated, while transcription factors such as *Otx2* and *Pou3f1*, along with the DNA methyltransferases *Dnmt3a* and *Dnmt3b,* are upregulated. These molecular events mark the transition of ES cells from naïve pluripotency toward a more lineage-restricted state known as the “formative” pluripotent state, which resembles the state of early post-implantation epiblast cells after specific induction (Smith, 2017; Kinoshita et al., 2021; Acampora et al., 2016; Auclair et al., 2014; Boroviak et al., 2014). In mice, epiblast stem cells (EpiSCs) are resistant to PGC induction. Naïve mES cells also cannot respond to germ cell inductive cues unless converted into epiblast-like cells (EpiLCs) within 24–48 h, which is essential for PGC specification (Kinoshita et al., 2021). During this transition, epiblast cells acquire the ability to initiate germline development and differentiate into primordial germ cells (PGCs). This process is critically regulated by genes such as *Tfap2c, Prdm1,* and *Prdm14*, which govern PGC fate and migration (Zhang et al., 2021; Yu et al., 2021). EpiLCs have distinct molecular and functional properties from naïve mES and epiblast stem cells. They resemble formative-phase pre-streak epiblast cells, exhibit intrinsic heterogeneity, and serve as a transient intermediate during pluripotent state transition. Subsequently, epiblast cells exhibit regional fate biases and molecular heterogeneity, entering the primed pluripotent state. Under specific activation of signaling pathways such as the Wnt and Nodal pathways and regulation by transcription factors such as *Fgf5* and *Tbxt*, these specialized cells initiate the transformation into the three germ layers (Nichols and Smith, 2009).

After establishment of potential pluripotent rabbit embryonic stem (rbES) cells in 1993 (Graves and Moreadith, 1993), most reported rbES cells have demonstrated characteristics including proliferation, self-renewal, *in vitro* differentiation into three germ layers, and teratoma formation; however, none have been confirmed to be capable of germline transmission (Graves and Moreadith, 1993). Currently, the reported stable state of rbES cells is the primed state (Afanassieff et al., 2020), which is similar to the situation observed in livestock species such as pigs and cattle, where stem cells are also generally maintained in a primed pluripotent state, with equally low chimerism efficiency in chimeric fetuses and no germline colonization achieved in any of these species (Fujishiro et al., 2013; Kawaguchi et al., 2015). Naïve-state cells have advantages over primed cells, including genetic manipulation and homogeneity, and further molecular regulatory pathways have been explored to control the transition of rbES cells from the primed to the naïve state (Afanassieff et al., 2020). Furthermore, Liu et al. obtained domed rbES cells with typical morphology and pluripotency markers from rabbit blastocysts using a 3i medium containing MEK, GSK3, and PKC inhibitors (Liu et al., 2019). Notably, Kobayashi et al. isolated rbES cells from early rabbit blastocysts by inhibiting the Wnt signaling pathway, and these cells could differentiate into primordial germ cell-like cells (PGCLCs) (Kobayashi et al., 2021). Recently, Nathalie’s team demonstrated that the VALGöX culture system, consisting of a PKC inhibitor and the Wnt pathway inhibitor XAV939, could initiate the transition of rabbit iPSCs from primed to naïve pluripotency, but failed to drive complete reprogramming and sustain *bona fide* naïve pluripotent characteristics. Furthermore, the team reprogrammed rabbit iPSCs with *KLF2*, *ERAS* and *PRMT6*, endowing the cells with efficient embryo colonization capacity (Pham et al., 2025).

ES cells are routinely cultured in media containing somatic feeder cell layers and fetal bovine serum (FBS); however, this system is susceptible to contamination by non-homologous cells, which compromises the accuracy of research outcomes (Hakala et al., 2009; Carpenter et al., 2004). Feeder-free culture allows the acquisition of highly homogeneous cell populations with relatively stable expression of pluripotency markers in embryonic stem cells (ESCs), making it ideal for exploring the mechanism by which small molecules regulate the maintenance of ESC pluripotency. Culture systems based on small molecules exhibit unique cellular biological characteristics. Although they differ somewhat from traditional culture models, they provide novel alternative schemes and optimization directions for stem cell culture (Xu et al., 2001; Tian et al., 2023). In a previous study, we successfully established rbES cell lines using an iFLY culture system with mouse embryonic fibroblasts (MEFs), and this system included LIF, basic fibroblast growth factor (bFGF), Noggin (a BMP pathway inhibitor) (Tancos et al., 2012), and the Rho kinase inhibitor Y-27632. These rbES cells displayed flattened colony morphology and expressed primed pluripotency markers (Du et al., 2015). In this study, we comprehensively explored the effects of multiple cytokines and small-molecule inhibitors that target distinct signaling pathways, including CHIR99021 (GSK-3α/β inhibitor) (Wu et al., 2015), PD0325901 (PD; MEK/ERK inhibitor) (Henderson et al., 2010), trichostatin A (TSA; histone deacetylase (HDAC) inhibitor) (Vigushin et al., 2001), 3-deazaneplanocin A (DZNep; EZH2 inhibitor) (Kikuchi et al., 2012), and IWP2 (Wnt inhibitor that targets Porcupine) (Mo et al., 2013; Jin et al., 2021). Specifically, using the iFLY culture system supplemented with bFGF, LIF, Noggin, and Y-27632, along with three small-molecule cellular inhibitory factors (IWP2, TSA, and DZNep, defined as 3C), we established the 3C system and successfully induced the transition of primed rbES cells toward a potentially formative pluripotent state. Transcriptomic analysis revealed that the 3C system drove rbES cell lines to adopt formative pluripotency features between naïve and primed states, which differed significantly from the primed characteristics observed under iFLY conditions. More importantly, the feeder-cell-free 3C system were successfully applied for *in vitro* differentiation into germline-specific PGCLCs, which have biological potential for further deriving functional oocytes from rbES cells.

## 2 Materials and methods

### 2.1 Maintaining rbES cell lines

Cryo-preserved passage 2 RbES cells were rapidly thawed in a 37 °C water bath. Upon complete thawing, twice the volume of iFLY culture medium was added (Du et al., 2015). Subsequently, the cell suspension was centrifuged at 300 ×g for 3 min. The supernatant was carefully removed, and the cell pellets were resuspended in iFLY medium. Suspended cells were subsequently seeded onto mitomycin C-treated MEF feeders (incubation with 10 μg/mL mitomycin C for 3 h) for ES cell maintenance. Using Mogengel Matrix (cat. no. 08277, Mogengel Bio, Xiamen, Fujian, China) coated culture dishes, we established the iFLY feeder-free culture condition. The iFLY base medium (BASE) (Du et al., 2015) consisted of KnockOut Dulbecco’s Modified Eagle Medium (DMEM; cat. no. 10819–018, Gibco, Grand Island, NY, USA) supplemented with 20% FBS (cat. no. SH30406.05, Hyclone, Logan, UT, USA), 1×non-essential amino acids (cat. no. M7145, Sigma-Aldrich, St. Louis, MO, USA), 1×β-mercaptoethanol (cat. no. ES-007-E, Millipore, St. Louis, MO, USA), 1× GlutaMax (cat. no. 35050–061, Gibco), and 100 U/mL penicillin-streptomycin (cat. no. SV30010, Hyclone). The iFLY medium consisted of BASE medium supplemented with 50 ng/mL Noggin (cat. no. 10267, SinoBiological, Beijing, China), 10 ng/mL bFGF (cat. no. 10014, SinoBiological), 10 ng/mL human leukemia inhibitory factor (hLIF; cat. no. 14890, SinoBiological), and 10 µM Y-27632 (It can be removed after 24 h after passaging; cat. no. T1870, TargetMol, Boston, MA, USA). Cells were passaged every 3–4 days. For routine subculture, cells were treated with TrypLE (cat. no. 12563029, Gibco) solution at room temperature for 3–5 min, and the digestion was neutralized with fresh culture medium. The cells were seeded at a split ratio of 1:3 to 1:5. Fresh iFLY medium was changed daily throughout the culture period.

### 2.2 Screening and optimizing feeder-free culture for rbES cells

Using a feeder-free culture system, we evaluated the effects of several cell factors at different concentrations on the pluripotency of rbES cells, including IWP2, a Wnt/β-catenin inhibitor (cat. no. T2702, TargetMol), at concentrations of 1, 5, and 10 μM; PD0325901, a MEK inhibitor (cat. no. T6189, TargetMol), at concentrations of 0.2, 1, and 3 μM; CHIR99021, a GSK-3β inhibitor (cat. no. T2310, TargetMol), at concentrations of 0.2, 1, and 3 μM; DZNep, an EZH2 methyltransferase inhibitor (cat. no. T6360, TargetMol), at concentrations of 10, 50, and 100 nM; and TSA, an HDAC inhibitor (cat. no. T6270, TargetMol), at concentrations of 5, 20, and 50 nM. We further examined the colony-forming capacity and expression levels of pluripotency-related genes in rbES cells following treatment with different small molecules. For the colony formation assay, 1000 rbES cells were seeded per well in 12-well plates and cultured in different conditioned media for 48 h. Alkaline phosphatase staining was then performed to evaluate colony-forming ability. The optimal combination of IWP2, TSA and DZNep was designated as the 3C feeder-free culture system. The basal 3C medium contained KnockOut DMEM, 20% FBS, 1× non-essential amino acids, 1× β-mercaptoethanol, 1× GlutaMax, and 100 U/mL penicillin–streptomycin, supplemented with 50 ng/mL Noggin, 10 ng/mL bFGF, 10 ng/mL hLIF, and 10 µM Y-27632 (which could be removed 24 h after cell passaging). The three small molecules were finally added at the working concentrations of 0.5 µM IWP2, 5 nM TSA, and 20 nM DZNep. All cell cultures were maintained at 37 °C in a humidified incubator with 5% CO_2_.

### 2.3 RNA isolation and library preparation

Total RNA was extracted using the RNA Isolator Total RNA extraction reagent (cat. no. R401-01, Vazyme, Nanjing, Jiangsu, China) according to the manufacturer’s protocol. The RNA purity and quantification were evaluated using the NanoDrop 2000 spectrophotometer (Thermo Scientific, USA). RNA integrity was assessed using the Agilent 2100 Bioanalyzer (Agilent Technologies, Santa Clara, CA, USA). Then, the libraries were constructed using the VAHTS Universal V10 RNA-seq Library Prep Kit (Premixed Version) according to the manufacturer’s instructions. Transcriptome sequencing and analysis were conducted by OE Biotech Co., Ltd. (Shanghai, China).

### 2.4 RNA sequencing and differentially expressed gene analysis

The libraries were sequenced on an Illumina Novaseq 6000 platform, and 150 bp paired-end reads were generated. Raw reads in the fastq format were first processed using fastp (Chen et al., 2018), and low-quality reads were removed to obtain the clean reads, which were mapped to the *Oryctolagus cuniculus* reference genome using HISAT2 (Kim et al., 2015). The fragments per kilobase of transcript per million mapped fragments value (Roberts et al., 2011) of each gene was calculated, and the read counts of each gene were obtained using HTSeq-count (Anders et al., 2015). Principal component analysis was performed using R (v 3.2.0) to evaluate the biological duplication of samples. Differential expression analysis was performed using DESeq2 (Love et al., 2014). A Q value < 0.05 and fold change >2 or <0.5 were set as the thresholds for significantly differentially expressed genes (DEGs). Hierarchical cluster analysis of DEGs was performed using R (v 3.2.0) to demonstrate the expression pattern of genes in different groups and samples.

Based on the hypergeometric distribution, Gene Ontology (GO) (The Gene Ontology Consortium, 2019), Kyoto Encyclopedia of Genes and Genomes (KEGG) pathway (Kanehisa et al., 2008), Reactome, and WikiPathways enrichment analyses of DEGs were performed to screen for significantly enriched terms using R (v 3.2.0). Gene set enrichment analysis (GSEA) was performed using GSEA software (Subramanian et al., 2005). The analysis used a predefined gene set, and the genes were ranked according to the degree of differential expression in the two types of samples. Then, we determined whether the predefined gene set was enriched at the top or bottom of the ranking list. R (v 3.2.0) was used to draw the column diagram, volcano plot, and heatmap.

### 2.5 Reverse transcription PCR analysis

Total RNA was extracted from rbES cells and ES cell-derived EBs using the RNA Isolator Total RNA extraction reagent. cDNA was synthesized using the HiScript III 1st Strand cDNA Synthesis Kit (cat. no. R312-01, Vazyme) following the manufacturer’s protocol. The 20 μL PCR reaction mixture contained 0.2 mM oligonucleotide primers, the cDNA template, Taq Master Mix (cat. no. P111, Vazyme), and nuclease-free water. After gentle vortexing and brief centrifugation, PCR was performed with an initial denaturation at 95 °C for 2 min, followed by 35 cycles of 95 °C for 30 s, 57 °C for 30 s, and 72 °C for 30 s. PCR products were analyzed by loading 10 μL onto a 2% agarose gel. All primer sequences are listed in Table 1.

**TABLE 1:** Primer sequences for PCR analysis.

Gene	Forward (5′-3′)	Reverse (5′-3′)	Size (bp)
*β-ACTIN*	ATG​CAG​AAG​GAG​ATC​ACC​GC	ACT​CCT​GCT​TGC​TGA​TCC​AC	148
*NANOG*	CAC​TGA​TGC​CCG​TGG​TGC​CC	AGC​GGA​GAG​GCG​GTG​TCT​GT	94
*POU5F1*	GCA​GCA​GAT​CAG​CCA​CAT​C	AAC​AGT​CAC​TGC​TTG​ATC​GTT​TG	107
*ZFP42*	GAC​GCT​GAC​TGA​ACG​CAT​ACC​A	CCA​CCC​TCC​TTT​CTC​ACG​ACC​A	262
*KLF17*	CAT​CTG​AAC​ATG​CGG​CAG​G	TGA​GTG​AAG​TGT​TGA​GTG​CTG​G	146
*LIN28B*	ACA​GCC​ACC​TGC​CAG​TTC​TCA	GGT​TCT​TCT​GGT​GCT​GCC​GAT​G	201
*OTX2*	CGC​CTT​ACG​CAG​TCA​ATG​G	GAGCGCTTCCAGCACATC	146
*FGF4*	GAG​CAG​CAA​GGG​AAA​ACT​ATA​CG	TAG​TTG​TTC​GGT​AGC​AGG​ATC​TC	81
*KLF4*	TAC​ACA​TGA​AGA​GGC​ACT​TTT​AAA​CC	TGT​GAA​AGG​CGA​CAA​ATA​CTG​AAC	86
*ESRRB*	CGT​GGA​GGC​CGC​CAG​AAG​TA	TCT​GGC​TCG​GCC​ACC​AAG​AG	131
*TBXT*	TCA​TGG​CTG​CGA​GAA​GTA​CC	ACA​GGC​TTG​GGT​ATT​GGC​TG	233
*NODAL*	CCG​CAA​GGT​CAA​GTT​CCA​GGT​G	AGC​CGC​ACT​CCT​CCA​CAA​TCA	296
*SOX2*	GAG​AAC​CCA​AGA​TGC​ACA​AC	CCG​TCT​CCG​ACA​AAA​GTT​TCC	79
*TFAP2C*	ACT​TTT​AAC​GGT​GGT​GGG​GG	CGG​CTT​CAT​TCG​GAA​TAC​CCT	81
*SOX17*	GAG​ACT​TGT​TCC​CCG​TCG​TT	GGC​GAT​ATC​TCC​AAA​ACT​GGT	75
*PRDM1*	TCC​CTG​TAA​CCA​GCA​CCA​CT	AAA​GCG​TTT​GGT​GTC​ACT​GT	87
*NANOS3*	GCC​TCC​GCC​TCT​ACC​TAG​C	TGA​CGT​CTT​CCA​AAG​CGC​AG	99
*NESTIN*	AGG​GGG​AAG​AGG​AAG​AGG​AGG​AG	TGC​TGC​AGC​CCG​TTC​ACC​ACA	394
*DESMIN*	AGC​AGG​AGA​TGA​TGG​AAT​AC	TCCAGCTTCCGGTAGG	281
*GATA6*	AAT​TCA​GAC​CAG​GAA​ACG​AAA​ACC	GGA​GTC​ATA​GGA​ACG​GAA​TTA​TTG​C	87
*GATA4*	CTC​AGA​AGG​CAG​AGA​GTG​TG	CCG​CAT​TGC​AAG​AGG​CCT​GG	321

### 2.6 Real-time qPCR analysis

Real-time qPCR was used to detect the expression of *POU5F1*, *Nanog*, *ZFP42*, *Klf17*, *LIN28B*, *OTX2*, *NODAL,* and *TBXT* in rbES cells cultured in the 3C and iFLY feeder-free systems. Total RNA was extracted from rbES cells using the RNA Isolator Total RNA extraction reagent. Using ABScript III RT Master Mix for qPCR (cat. no. RK20429, Abclonal), 1 μg of total RNA was reverse-transcribed into cDNA according to the instructions. qPCR was performed using Genious 2X SYBR Green Fast qPCR Mix (cat. no. RK21207, Abclonal) on a Stepone Plus 96-Well Thermal Cycler (Applied Thermo, USA). The qPCR conditions were as follows: 95 °C for 3 min, followed by 40 cycles of 95 °C for 5 s and 60 °C for 30 s. The gene expression levels were analyzed using the 2^−ΔΔCT^ method with *β-ACTIN* as the control. All primer sequences are listed in Table 1.

### 2.7 Total protein extraction and Western blotting

Total protein was extracted from rabbit embryonic stem (rbES) cells using RIPA lysis buffer (Cat. No. C500005, Sangon Biotech, China). In brief, cells were rinsed three times with ice-cold DPBS, followed by the addition of an appropriate volume of lysis buffer. After incubation on ice for 3–5 min, cells were scraped and transferred into prechilled 1.5 mL centrifuge tubes. Samples were fully lysed by vigorous vortexing for 10 min and then centrifuged at 13,400 × g and 4 °C for 15 min. The supernatants were collected, and protein concentrations were determined using a BCA protein assay kit (Cat. No. E112-01, Vazyme, China).

For protein denaturation, 6 μg of total protein was mixed with protein loading buffer (cat. no. RM00001, ABclonal, China) and heated at 100 °C for 10 min. Protein samples were then separated by SDS-PAGE, with the separation gel concentration adjusted according to the molecular weight of target proteins. After electrophoresis, proteins were electrotransferred onto PVDF membranes (cat. no. 03010040001, Roche, Basel, Switzerland). The membranes were blocked with 5% non-fat milk prepared in TBST (TBS containing 0.1% Tween-20) for 1 h at room temperature. After washing with TBST, the membranes were incubated overnight at 4 °C with primary antibodies: rabbit anti-H3K27me3 (cat. no. A22396, ABclonal, Wuhan, China; 1:10,000), rabbit anti-H3K27ac (cat. no. WH427276, ABclonal, Wuhan, China; 1:10,000), rabbit anti-H3K4me3 (cat. no. A22146, ABclonal, Wuhan, China; 1:5000), and rabbit anti-Histone H3 (cat. no. 17168-1-AP, Proteintech Group, Rosemont, IL, USA; 1:10,000). After three washes with TBST, the membranes were incubated with HRP-conjugated goat anti-rabbit secondary antibody (1:10,000, cat. no. BS13278, Bioworld, USA) for 2 h at room temperature. Protein bands were detected with a SuperPico ECL chemiluminescence kit (cat. no. E422-01, Vazyme, China) and imaged using a Tanon 4600 imaging system. Band intensity was quantified with ImageJ software, and total H3 was used as the internal reference for data normalization.

### 2.8 Alkaline phosphatase staining

Alkaline phosphatase staining was performed using a BCIP/NBT Alkaline Phosphatase Staining Kit (cat. no. C3250S, Beyotime, Shanghai, China). The procedure was as follows: Cells were fixed with 4% paraformaldehyde (PFA) for 15 min at room temperature after being washed three times with Dulbecco’s phosphate-buffered saline (DPBS). The fixative was discarded, and the cells were again washed three times with DPBS. Then, 1× BCIP/NBT chromogenic solution was prepared according to the kit instructions and added to the cells for incubation in the dark at room temperature for 30–40 min, with the staining process monitored. The reaction was terminated, and the cells were thoroughly washed three times with DPBS, after which a small amount of DPBS was left to cover the cell layer. Images were captured using an Olympus IX73 microscope and DP74 camera.

### 2.9 Karyotyping

Chromosome karyotype analysis was carried out as previously described (Liu et al., 2019). Cells were treated with 100 ng/mL colchicine (Cat. No. ST1173, Beyotime) and incubated at 37 °C under 5% CO_2_ for 3 h. After TrypLE digestion, cell pellets were harvested and treated with 0.075 M KCl hypotonic solution for 20 min. Following centrifugation at 300 *g* for 5 min, cells were pre-fixed with 1 mL fresh fixative (methanol:glacial acetic acid = 3:1, v/v) for 5 min. After another centrifugation at 300 *g* for 5 min, cells were fixed in ice-cold fixative for 30 min. Subsequently, cells were resuspended in 0.5 mL fresh fixative, dropped and dispersed onto ice-cold slides. Air-dried slides were stained with 10% Giemsa solution (in 10 mM potassium phosphate buffer, pH 6.8) for 30 min. Finally, microscopic observation and image capture were performed, and a total of 50 metaphase chromosome spreads were randomly selected for karyotype analysis.

### 2.10 Immunofluorescence staining

The cells were fixed with 4% PFA (cat. no. 16005, Sigma-Aldrich) at room temperature for 10 min and then washed three times with DPBS (cat. no. 14190–144, Gibco) for 5 min each. Next, the cells were permeabilized with DPBS containing 0.2% Triton X-100 (cat. no. 9002–93–1, Solarbio, Beijing, China) for 10 min and blocked with DPBS containing 5% bovine serum albumin (cat. no. BSA-embryo 02, Renova Life, Maryland, USA) at room temperature for 1 h. The samples were then incubated with the following primary antibodies overnight at 4 °C: rabbit anti-SOX17 (cat. no. AF 1924, R&D, Minneapolis, MN, USA; 1:200), rabbit anti-TBXT (cat. no. Ab5078, Abclonal; 1:200), rabbit anti-βIII-Tubulin (cat. no. A17913, Abclonal; 1:200), goat anti-hSOX2 (cat. no. AF 2018, R&D; 1:200), rabbit anti-GATA4 (cat. no. A4306, Abclonal; 1:200), goat anti-TFAP2C (cat. no. AF5059, R&D; 1:200), mouse anti-POU5F1 (cat. no. Sc5279, Santa Cruz; 1:50), and rabbit anti-OTX2 (cat. no. A4351, Abclonal; 1:200). After three washes with DPBS, the samples were incubated with the following secondary antibodies at room temperature for 1 h: Cy3-conjugated goat anti-rabbit IgG H + L (cat. no. AS007, Abclonal; 1:200), Cy3-conjugated rabbit anti-goat IgG H + L (cat. no. AS015, Abclonal; 1:200), and ABflo®488 goat anti-mouse IgG H + L (cat. no. AS037, Abclonal; 1:200). DNA was stained with Hoechst 33,342 (cat. no. BD5011, Bioworld Technology Bloomington, MN, USA). Images were acquired using an Olympus IX73 microscope and DP74 camera.

### 2.11 Embryoid body (EB) formation assay and *in vitro* three germ layer differentiation of 3C system cultured rbES cells

rbES cells were dissociated into a single-cell suspension using TrypLE Express (cat. no. 12604–021, Gibco). The cells were resuspended in a specialized medium prepared with DMEM/F12 (cat. no. C11330500, Gibco) supplemented with 20% FBS, 10 ng/mL bFGF, 10 ng/mL hLIF, and 10 µM Y-27632. This cell suspension was then transferred to a low-adhesion culture dish. After 7 days in suspension culture, the EBs were used for lineage-specific gene RT-PCR detection and immunostaining experiments.

Based on the differentiation protocol for rabbit pluripotent stem cells established by Toshihiro Kobayashi, rbPSCs were directionally differentiated into definitive endoderm (DE), lateral mesoderm (LM) and neuroectoderm (NE) in aFB27 basal medium supplemented with specific inducers (Kobayashi et al., 2021). The aFB27 basal medium was prepared with Advanced DMEM/F12 supplemented with 1% B27 supplement, 0.1 mM non-essential amino acids (NEAA), 100 U/mL penicillin, 0.1 mg/mL streptomycin, 2 mM L-glutamine and 0.1% polyvinyl alcohol (PVA). For DE differentiation, single-cell suspensions of digested rbPSCs were seeded onto vitronectin-coated 12-well plates at a density of 2 × 10^5^ cells per well and cultured in mesendoderm (ME) induction medium (aFB27 medium containing 100 ng/mL Activin-A, 3 μM CHIR99021, 50 nM PI103 (cat. no. T6143, TargetMol) and 10 μM ROCK inhibitor Y27632) for 24 h to generate mesendoderm progenitor cells; after ME induction, cells were rinsed once with PBS and cultured in DE induction medium (aFB27 medium supplemented with 100 ng/mL Activin-A, 0.5 μM LDN193189 (BMP inhibitor; cat. no. T1935, TargetMol) and 2.5 μM IWR1) for another 2 days with daily medium renewal, and then harvested for the detection of endodermal markers. For LM differentiation, rbPSCs were seeded and induced into mesendoderm progenitors via the same ME induction procedure as DE differentiation; following one wash with PBS, the medium was replaced with LM induction medium (aFB27 medium supplemented with 100 ng/mL BMP4, 2.5 μM IWR1 and 10 μM TGF-β inhibitor SB431542 (cat. no. T1726, TargetMol)), and cells were continuously cultured for 2 days with daily medium change, then collected to detect mesodermal signature genes and proteins. For NE differentiation, dissociated rbPSCs were seeded at 2 × 10^5^ cells per well on vitronectin-precoated 12-well plates and cultured directly in NE induction medium (aFB27 medium supplemented with 10 μM SB431542, 0.5 μM LDN193189 and 10 μM Y27632) for 72 h with fresh medium replaced every day, and cells were collected for the identification of ectodermal markers after induction.

### 2.12 *In vitro* induction and derivation of germline-specific PGCLCs from 3C rbES cells

For PGCLC induction, rbES cells were dissociated into a single-cell suspension using TrypLE Express when they reached 80% confluence. The cells were then centrifuged at 300 *g* for 3 min to collect the pellet, which was subsequently resuspended in PGCLC induction medium. The cells were seeded into a 96-well, low-adhesion, U-shaped plate (cat. no. 174925, Thermo Fisher Scientific, Waltham, MA, USA) at a density of 5 × 10^4^ cells per well, with 200 µL of PGCLC induction medium added to each well. The PGCLC induction medium was formulated as follows: G-MEM (cat. no. 11710–035, Gibco) was supplemented with 1× penicillin-streptomycin, 1× GlutaMax, 1 mM sodium pyruvate (cat. no. 11360–070, Gibco), 20% KOSR (cat. no. 10828, Gibco), 1× non-essential amino acids, 1× β-mercaptoethanol, 10 µM Y-27632 (continuously present during culture), 3 µM CHIR99021, 10 ng/mL hLIF, 50 ng/mL BMP4 (cat. no. 10609, SinoBiological), 50 ng/mL epidermal growth factor (EGF; cat. no. 10605, SinoBiological), and 50 ng/mL stem cell factor (SCF; cat. no. 10451, SinoBiological). During the culture period, rbES cells formed cell aggregates. The culture medium was refreshed by replacing 50% of the medium with fresh medium daily, and the cells were cultured for 3.5 days at 37 °C, 5% CO_2_, and constant humidity. For immunofluorescence identification, after the cell aggregates were cultured for 3.5 days, they were dissociated into a single-cell suspension using TrypLE Express and seeded into a gelatin-coated 24-well plate at a density of 5 × 10^4^ cells per well. After 6 h of culture in PGCLC induction medium, the cells were fixed with 4% PFA for subsequent antibody detection. In addition, qPCR was employed to detect the expression of *SOX17*, *NANOS3*, *PRDM1*, and *TFAP2C* in rbES cells after 3.5 days of induction. Furthermore, the cell aggregates were further cultured to day 10, and whole-aggregate immunofluorescence staining was performed to detect the expression of VASA (cat. no. A15624, Abclonal; 1:200) and POU5F1.

### 2.13 Data access

The raw sequence data reported in this paper have been deposited in the Genome Sequence Archive (Chen et al., 2021) in National Genomics Data Center (C NCB-NGDC Members and Partners, 2022), China National Center for Bioinformation/Beijing Institute of Genomics, Chinese Academy of Sciences (GSA: CRA026197 and CRA041789) that are publicly accessible at https://ngdc.cncb.ac.cn/gsa.

### 2.14 Statistical analysis

All experiments were repeated at least three times. rbES cells cultured under both 3C and iFLY conditions were derived from the same cell line to exclude variations caused by different cell lines. Data were analyzed using SPSS version 18.0 (SPSS, Inc., Chicago, IL, USA) and are presented as mean ± standard deviation (SD). Comparisons between two groups were made by Student’s two-tailed t-tests. Statistical significance was defined as P < 0.05. Image analysis was performed using ImageJ (Bethesda, USA). Figures were generated using GraphPad Prism 10 (La Jolla, USA).

## 3 Results

### 3.1 Identification of the pluripotency of rbES cells derived from 3C feeder-free culture

**FIGURE 1:**
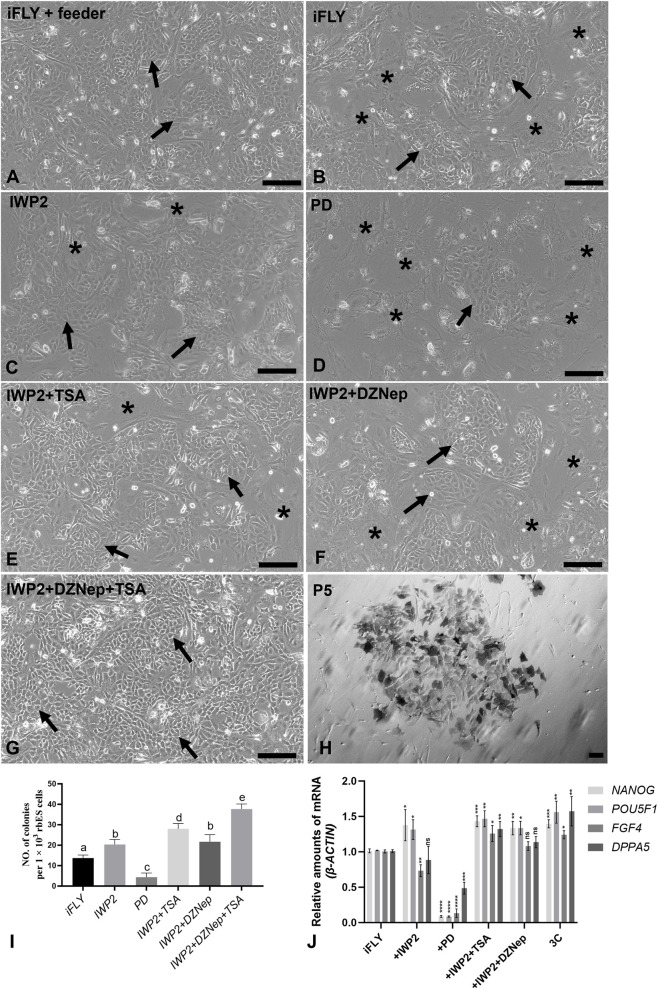
Testing rbES cell culture conditions in the iFLY feeder-free system. **(A)** Flat colonies of rbES cells derived from the iFLY system grown on MEFs. **(B)** Flat colonies of rbES cells derived from the iFLY feeder-free system. **(C)** Cell culture using the iFLY feeder-free system with 0.5 μM IWP2 for 2 days reduced cell differentiation. **(D)** Addition of 1 μM PD0325901 to the iFLY feeder-free system for 2 days resulted in severe rbES cell differentiation and death. **(E)** After 2 days of culture using the iFLY feeder-free system with 0.5 μM IWP2 and 5 nM TSA, cell differentiation decreased and rbES cell clone formation increased. **(F)** Culture using the iFLY feeder-free system with 0.5 μM IWP2 and 20 nM DZNep for 2 days decreased differentiation and increased rbES cell clones. **(G)** Upon exposure to 0 0.5 μM IWP2, 20 nM DZNep, and 5 nM TSA (3C system) for 2 days, cells cultured with the iFLY feeder-free system exhibited reduced differentiation and an increase in rbES cell clones. Black arrows indicate rbES cell clones, and asterisks (*) indicate differentiated cells. **(H)** rbES cells from the 3C system showed strong alkaline phosphatase activity. **(I)** The number of rbES cell colonies after 2 days of culture with different small molecules is presented as the mean ± SD (n = 3). The letters a, b, c, d, and e denote significant differences between groups (*P* < 0.05). **(J)** Pluripotency gene expression levels in rbES cells cultured with different small molecule combinations. An independent sample t-test was used to compare each group with its respective matched control; *P < 0.05, **P < 0.01, ***P < 0.001, ****P < 0.0001. Scale bar = 100 μm.

The rbESCs used in this study were thawed at passage 2, and all subsequent experiments were performed between passages 8 and 25 to ensure cell stability. These cells were stably maintained on feeder cells in iFLY medium, which is consistent with the culture conditions described in our previous studies (Du et al., 2015). On the basis of this established iFLY rbES cell system (Figure 1A), we explored an optimal feeder-free culture strategy by eliminating MEF feeder cell interference. Mogengel Matrix was substituted for conventional MEF feeders, with the addition of 10 ng/mL bFGF, 10 ng/mL hLIF, 50 ng/mL Noggin, and 10 μM Y-27632 to the medium to establish the iFLY feeder-free system. However, rbES cells cultured in feeder-free medium displayed retardation of proliferation and flattened morphological phenotypes (Figure 1B), indicating spontaneous and progressive cell differentiation.

After adding the Wnt/β-catenin signaling pathway inhibitor IWP2, the cell proliferation rate and clonogenic capacity were significantly enhanced compared with those of the iFLY feeder-free control (IWP2) (Figure 1C); however, this was not sufficient to suppress the differentiation process. In contrast, when different concentrations of the Wnt pathway activator CHIR99021 (0.2, 1.0, and 3 µM) were added to the iFLY feeder-free system, the pluripotency marker POU5F1 was expressed with 0.2 μM but completely suppressed with 1 and 3 μM CHIR99021 (Supplementary Figures S1A-C). Notably, addition of the MEK/ERK pathway inhibitor PD caused a dose-dependent loss of cell pluripotency (Figure 1D) at concentrations of 0.2, 1.0, and 3 μM (Supplementary Figures S1D-F), which is consistent with prior studies on bovine ES cell cultures (Zhao et al., 2021). Epigenetic modifications involved in regulation of ES cell gene expression include histone methylation and histone acetylation (Cao and Zhang, 2004; Saraiv et al., 2010). We further optimized the experiment by combining either IWP2 with the HDAC inhibitor TSA (5 nM) (Figure 1E, IWP2+TSA) or with the histone methyltransferase EZH2 inhibitor DZNep (20 nM) (Figure 1F, IWP2+DZNep). The combination of IWP2 and TSA significantly improved clonogenic efficiency compared with that in the IWP2 or PD group (*P* < 0.05) (Figure 1I), but differentiated cells were still observed at the edges of colonies (Figure 1E). A slight increase in rbES cells was observed in the IWP2+DZNep group compared with the level in the PD group (Figure 1F), but it was not different from that of IWP2 treatment alone (Figure 1I). When the three small-molecule compounds (3C: IWP2+DZNep + TSA) were applied together, rbES cell colony growth was significantly improved, typical “nest-like” colony structures were obvious, and the proportion of differentiated cells between adjacent colonies was markedly reduced (Figure 1G). Moreover, alkaline phosphatase-positive (Figure 1H) and POU5F1- and SOX2-positive cell colonies (Figure 3C) were clearly observed, and the rbES cell colony ratio was the highest among all treatments (Figure 1I; Supplementary Figures S4B, *P* < 0.05). RT-qPCR analysis revealed that IWP2 treatment effectively enhanced the pluripotency of rabbit embryonic stem cells (rbESCs). Upon further supplementation with 5 nM TSA in the IWP2-conditioned medium, the expression of the core pluripotency genes *NANOG* and *POU5F1* was significantly elevated, along with obvious upregulation of *FGF4* and *DPPA5*. Conversely, exposure to PD0325901 markedly compromised the pluripotent state of rbESCs (Figure 1J).

**FIGURE 2:**
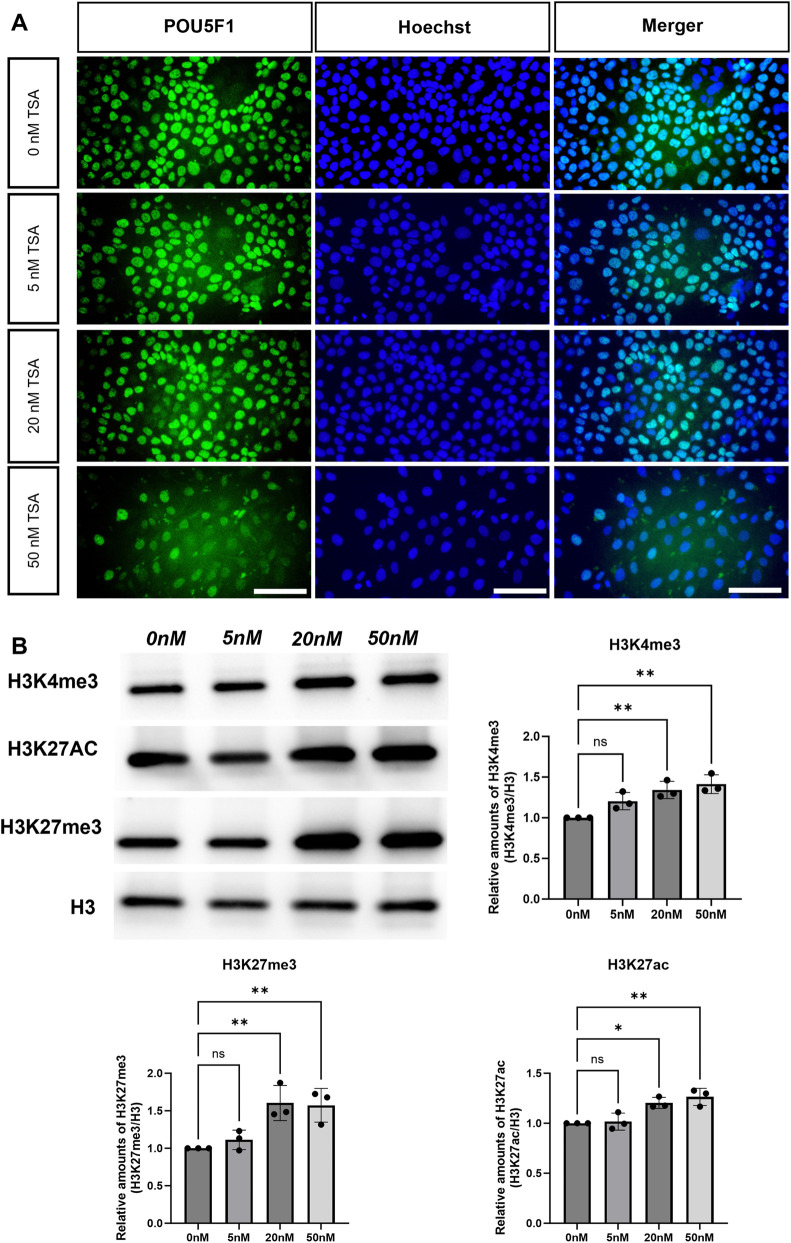
Effects of TSA gradient on pluripotency and histone modifications in rbES cells. **(A)** Immunofluorescence staining of pluripotency marker POU5F1 in rbES cultured in IFLY + IWP2 system and treated with gradient concentrations of TSA (0, 5, 20, 50 nM). **(B)** Western blot analysis of H3K27ac, H3K27me3 and H3K4me3 in rbES cells cultured in IFLY plus IWP2 system treated with 0, 5, 20 and 50 nM TSA. All histone modification band intensities were normalized to total H3. One-way ANOVA followed by Dunnett’s multiple comparison test was used to compare each TSA group with the 0 nM control; *P < 0.05, **P < 0.01; ns, no significant difference.

To verify the regulatory effect of TSA on pluripotency maintenance of rabbit embryonic stem cells (rbESCs), rbESCs cultured in the IWP2-containing iFLY system were treated with a gradient of TSA concentrations for 24 h. The results showed that high concentrations of TSA (20 nM and 50 nM) disrupted the pluripotent state of rbESCs and markedly downregulated the expression of POU5F1, whereas no significant changes were observed in the low-concentration (5 nM) TSA group (Figure 2A). We further detected the protein levels of histone modifications including H3K27me3, H3K27ac and H3K4me3. The results revealed that high-concentration TSA treatment significantly elevated the enrichment levels of H3K27me3, H3K27ac and H3K4me3 (Figure 2B).

**FIGURE 3:**
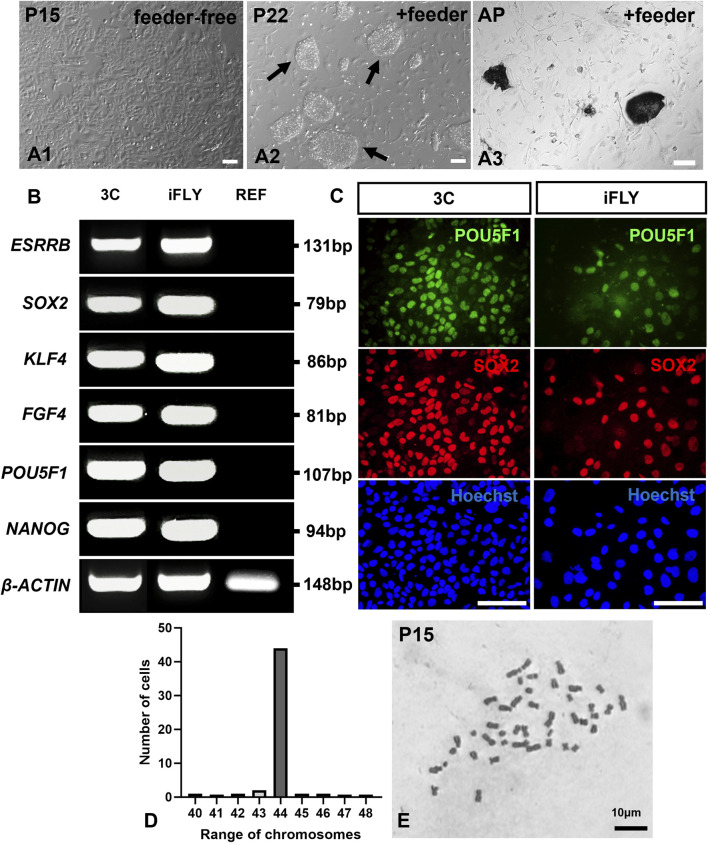
Characterization and Identification of rbES Cells. **(A)** Brightfield morphology of rbES cells cultured for 15 passages in the 3C feeder-free system. Passage 22 rbES cells derived from the 3C system were seeded on MEF feeder layers, formed compact colonies (arrowed), and exhibited positive AP staining. **(B)** Expression of pluripotency markers by RT-PCR using total RNA extracted from 3C and iFLY feeder_free culture systems with primers for *ESRRB*, *SOX2*, *KLF4*, *FGF4*, *POU5F1*, *NANOG*, and *β_ACTIN*. Rabbit ear fibroblasts (REF) were used as the somatic cell control. **(C)** rbES cells from the 3C and iFLY feeder-free culture systems exhibited positive immunofluorescent signals for POU5F1 and SOX2. **(D)** Chromosome analysis of rbES cells at passage 15. The modal chromosome number was 44, and the rate of normal karyotype was 88%. **(E)** Metaphase chromosomes were micrographed after Giemsa staining. Scale bar=100 μm, unless otherwise specified.

Following long-term *in vitro* cultivation, rbESCs maintained normal cellular morphology up to passage 15. When 3C-cultured rbESCs were seeded onto MEF feeder layers, they formed typical stem cell colonies and displayed strong positive alkaline phosphatase activity (Figure 3A). At passage 4, RT-PCR analysis was performed to examine the expression of representative pluripotency marker genes in rbES cells cultured in the 3C and iFLY systems (Figure 3B). The results revealed that *POU5F1*, *SOX2*, *NANOG*, *FGF4*, *KLF4* and *ESRRB* were all highly expressed in both culture conditions, whereas no expression of these genes was detected in rabbit ear epithelial cells. Notably, cells cultured in the 3C system maintained a normal karyotype after long-term culture up to passage 15 (Figures 3D,E).

### 3.2 Expression of naïve and formative pluripotency genes in rbES cells produced using the 3C feeder-free system

**FIGURE 4:**
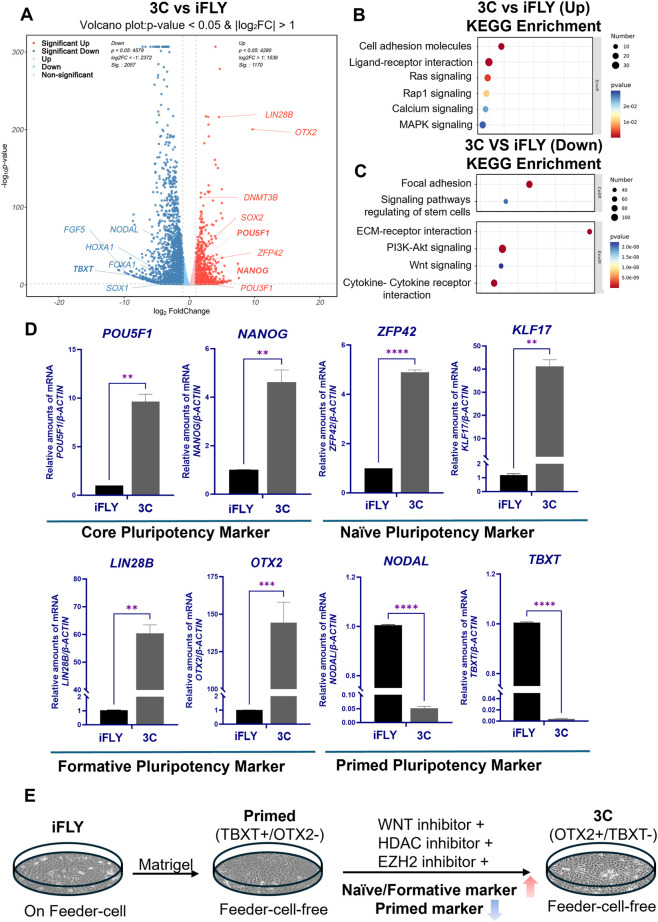
Transcriptome analysis and gene expression. **(A)** Volcano plot of gene expression in the 3C and iFLY systems. Blue dots mark significantly downregulated genes; red dots mark upregulated genes. Naïve pluripotency markers (e.g., *ZFP42*) and formative pluripotency markers (e.g., *OTX2* and *LIN28B*) were upregulated in the 3C system, while primed pluripotency markers (e.g., *NODAL*, *HOXA1*, and *TBXT*) were downregulated. **(B)** KEGG analysis showed significant upregulation of the Ras and MAPK pathways in 3C system-derived rbES cells. The upregulated genes were primarily enriched in pathways related to cell communication and adhesion, including cell adhesion molecules, ligand-receptor interaction, Rap1 signaling, and MAPK signaling, suggesting enhanced cell-cell interaction and environmental responsiveness in the 3C state. **(C)** KEGG analysis revealed significant downregulation of the PI3K-Akt and Wnt pathways in 3C system-derived rbES cells. **(D)** RT-qPCR showed that in the 3C system, *POU5F1*, *NANOG*, *ZFP42*, *KLF17*, *LIN28B*, and *OTX2* were significantly upregulated, while *NODAL* and *TBXT* were significantly downregulated compared with their levels in cells from the iFLY system. *β-ACTIN* was used to normalize the data, which are presented as the mean ± SD. Independent samples t-tests were used for statistical analysis. **, ***, and **** indicate *P* < 0.01, <0.001 and <0.0001, respectively. **(E)** Schematic illustrating the establishment of the feeder-free 3C culture system for rbES cells.

**FIGURE 5:**
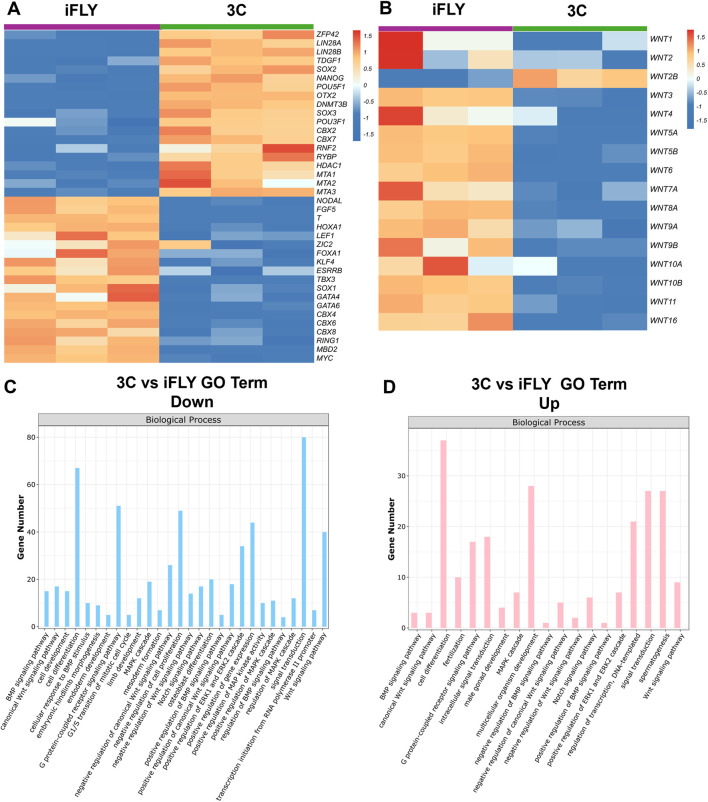
Transcriptional map of rbES cells. **(A)** Heatmap showing the expression levels of 38 genes reported to be associated with pluripotency and differentiation in the early embryonic lineages of rodents and primates. **(B)** Heatmap showing downregulation of Wnt signaling-related ligand genes in 3C system-cultured rbES cells. **(C,D)** GO biological process enrichment analysis identified genes in cell development-related signaling pathways, including the BMP, Wnt, and Notch pathways, that showed increased (red) or decreased (blue) expression.

RNA-Seq and RT-qPCR analyses clearly demonstrated that the core transcription factors *SOX2*, *POU5F1,* and *NANOG* were highly expressed under the 3C system (Figures 4A,D). Naïve-state markers such as *KLF17* and *ZFP42* showed significant upregulation (Figures 4A,D), and elevated expression of formative-state marker genes (*OTX2*, *LIN28B*, and *DNMT3B*) was observed (Figures 4A,D). Conversely, primed marker genes (*NODAL* and *TBXT*) (Figures 4A,D) and differentiation-related genes (*GATA4*, *GATA6*, and *FOXA1*) were markedly downregulated in the 3C system (Figure 5A). We compared the transcriptomic profiles of embryonic day 3.5 ICM (E3.5) with those of rbESCs cultured in the 3C and iFLY systems. The heatmap showed that lineage-specific differentiation markers, including endodermal genes *FOXA1*, *GATA4*, and *GATA6*, the neuroectodermal marker *SOX1*, and mesodermal genes *T* and *HOXA1*, were strongly repressed in both the ICM and 3C groups but significantly upregulated in the iFLY group. Similarly, epigenetic regulators of developmental genes (e.g., Polycomb repressive complex components *CBX4*, *CBX8*, and *RNF2*) and formative-state markers exhibited an expression pattern consistent with the ICM in 3C-cultured cells, whereas these differentiation-related genes were markedly upregulated in the iFLY group (Supplementary Figures S3). Furthermore, ligand genes associated with the Wnt signaling pathway displayed significant downregulation in 3C cultures (Figure 5B). KEGG pathway enrichment analysis of DEGs between the 3C and iFLY feeder-free systems revealed upregulated expression of genes associated with the Ras, Rap1, calcium, and MAPK signaling pathways in the 3C system (Figure 4B). The Ras signaling pathway regulates cellular proliferation, differentiation, survival, and migration (Yang and Wu, 2024). Rap1, a small GTPase of the Ras superfamily, is involved in processes such as cell adhesion, migration, and survival (Rothenberg et al., 2023). The upregulation of these pathways indicated that ES cells in the 3C system were in a state of active proliferation or differentiation. Conversely, genes linked to the PI3K-Akt and Wnt signaling pathways were downregulated in the 3C system (Figure 4C). GO enrichment analysis further demonstrated reduced numbers of cell differentiation-related genes and decreased expression of BMP signaling pathway-associated genes in the 3C system (Figures 5C,D). Likewise, using available antibodies in our laboratory, immunofluorescence analysis revealed distinct and strong OTX2 expression in rbES cells cultured under the 3C system but not for the iFLY system (Supplementary Figures S2A,B), and either minimal or undetectable TBXT signals (priming marker) were observed. In contrast, TBXT was highly expressed in rbES cells from the iFLY system (Figure 4E; Supplementary Figures S2C,D).

### 3.3 rbES cells can efficiently differentiate into all three germ layers *in vitro*

**FIGURE 6:**
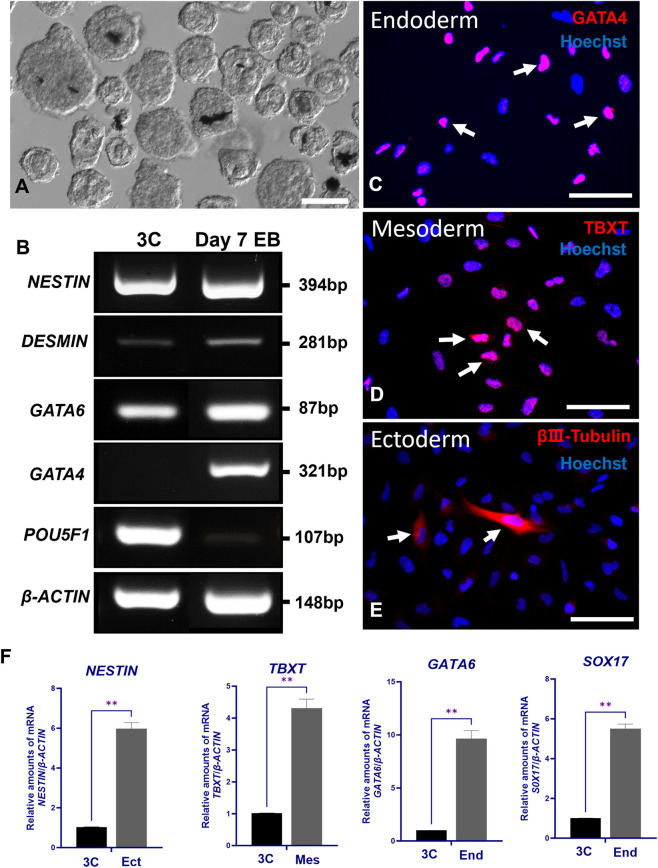
Embryoid body formation and trilineage differentiation potential of rbES cells derived from the 3C system. **(A)** EBs formed after 72 h of culture in low-adhesion cell culture dishes. **(B)** RT-PCR analysis of three germ layer-specific genes expressed in rbES cell-derived EBs. The endoderm markers *GATA4* and *GATA6*, mesoderm marker *DESMIN*, and ectoderm marker *NESTIN* were all expressed. **(C–E)** Immunofluorescence staining of differentiation markers for the three germ layers in EBs derived from rbES cells. Arrows indicate cells that differentiated into the three germ layers. Scale bar = 100 μm. **(F)** RT-qPCR analysis of lineage-specific gene expression in rbESCs following directed trilineage differentiation. Genes specific to the ectoderm (NESTIN), mesoderm (TBXT), and endoderm (GATA6, SOX17) were examined. Data were normalized to *β-ACTIN*. Error bars represent the mean ± SD (n = 3). Statistical analysis was performed using an independent samples t-test. ** indicate *P* < 0.01.

We investigated the *in vitro* differentiation capacity of the 3C cell line. In suspension culture, the 3C cell line formed EBs (Figure 6A). After 7 days of culture, the EBs were collected and analyzed by RT-PCR, which revealed expression of marker genes for all three germ layers: *GATA4* and *GATA6* (endoderm), *DESMIN* (mesoderm), and *NESTIN* (ectoderm), with no detectable expression of the pluripotency marker *POU5F1* (Figure 6B). The 7-day EBs were dissociated into single cells and plated for adherent culture for 24 h. Immunofluorescence staining confirmed the presence of endoderm-derived GATA4-expressing cells, mesoderm-derived TBXT-expressing cells, and ectoderm-derived neuronal βIII-tubulin-expressing cells in the adherent cultures (Figures 6C–E). To further validate the three-germ-layer differentiation potential of rbESCs cultured in the 3C system, we performed directed differentiation assays toward each germ lineage. RT-qPCR analysis showed that the ectodermal marker NESTIN, mesodermal marker TBXT, and endodermal markers GATA6 and SOX17 were significantly upregulated in the corresponding differentiated groups (Figure 6F), confirming that these cells retain the capacity to initiate differentiation programs toward all three germ lineages.

### 3.4 Germline specific induction and differentiation of PGCLCs from 3C rbES *in vitro*

**FIGURE 7:**
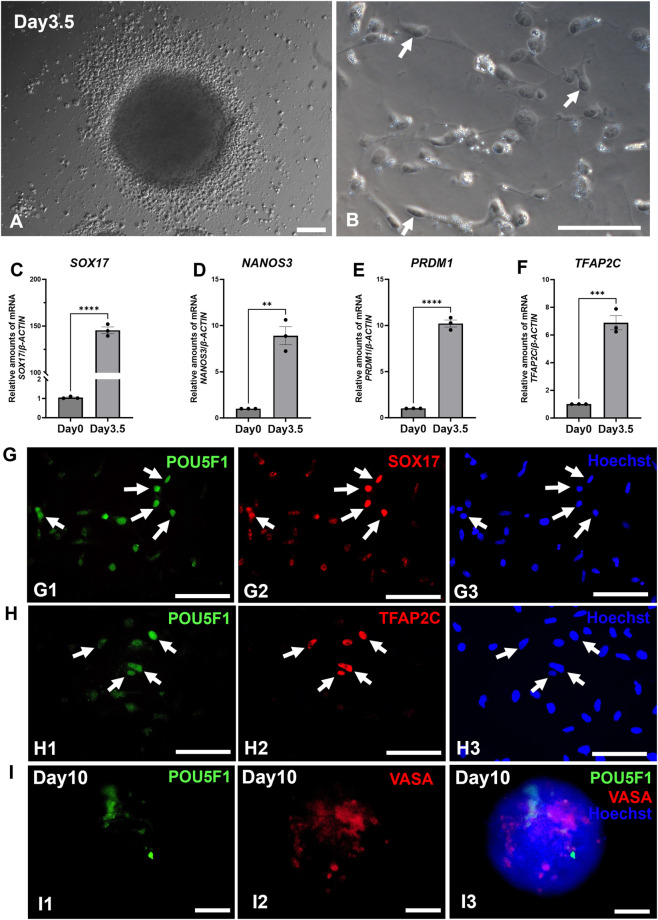
The induction of rabbit PGCLCs from rbES cells. **(A)** EBs formed as spheres after 24 h of directed induction in low-adhesion U-shaped dishes. **(B)** After 84 h of induction, the spheres were digested and adherently cultured for cell immunofluorescence identification. **(C–F)** RT-qPCR analysis showing significant upregulation of PGC marker genes (*SOX17*, *NANOS3*, *PRDM1*, and *TFAP2C*) in cells after 84 h of induction. Data were normalized to *β-ACTIN* and analyzed using independent sample t-tests. Error bars represent the mean ± SD. **, ***, and **** indicate *P* < 0.01, <0.001, and <0.0001, respectively. **(G,H)** Immunofluorescence staining showing positive signals for POU5F1, SOX17, and TFAP2C, indicating the presence of early PGCLCs. **(I)** Whole-mount immunostaining of day 10 PGC-induced EBs derived from 3C cultured rbES cells revealed positive signals for both VASA and POU5F1. Arrows indicate PGCLCs. Scale bar = 100 μm.

We directly induced rbES cells derived from the 3C system to form PGCLCs *in vitro*. Cells were seeded in low-adhesion 96-well plates and stimulated with BMP4, EGF, and SCF for 3.5 days. During this period, EB formed (Figure 7A), and transcript levels of the PGC-specific genes *SOX17*, *NANOS3*, *PRDM1*, and *TFAP2C* were markedly upregulated (Figures 7C–F). Compared with cells cultured in the iFLY system, rbES cells maintained in the 3C system exhibited a more rapid response to BMP4 signaling during germline differentiation. Under PGCLC-inducing conditions, the expression levels of PGC-specific genes *SOX17*, *NANOS3*, *PRDM1*, and *TFAP2C* were significantly higher in the 3C group than in the iFLY group (Supplementary Figures S4C). These embryoid bodies collected at day 3.5 were dissociated into single cells and subjected to immunofluorescence staining, which identified SOX17^+^ and TFAP2C^+^ cells (Figures 7G,H). By day 10 of induction, VASA expression was further detected in the cell aggregates, confirming that the early PGCLCs had progressed toward a more mature germ cell stage (Figure 7I).

These results indicated that by using the 3C system, rbES cells could directly respond to germline-specific signaling pathways by differentiating into PGCLCs.

## 4 Discussion

In this study, we demonstrated that rbES cells at an intermediate state between the naïve and primed pluripotency could be successfully derived from previously established iFLY-primed rbES cells (Du et al., 2015) by inducing with three small molecule compounds, IWP2 (Wnt inhibitor), TSA (HDAC inhibitor), and DZNep (EZH2/PRC2 inhibitor) under an MEF somatic feeder-free system (3C system: IWP2+DZNep + TSA) (Figure 4E). The feeder-free 3C system provides a defined condition that eliminates cellular and molecular interference from exogenous somatic cells (Ludwig et al., 2006) and further makes it possible to explore inherent molecular events and regulatory mechanisms in stem cells (Chen et al., 2011). We report a non-transgenic and chemically-defined culture system that can robustly promote the pluripotent transition of rbES cells from the primed to the formative state. A significant increase in the rbES cell nest-like colony ratio was achieved under the feeder-free 3C system. Under feeder-free 3C conditions, rbES cells displayed a markedly stabilized pluripotent identity. Collectively, the findings indicate that the 3C system efficiently promotes the primed-to-naïve transition in rbES cells, likely through selectively expanding heterogeneous cell subpopulations at different pluripotent stages (Bernemann et al., 2011; Tsakiridis et al., 2014). Comparative transcriptomic profiling and RT-qPCR analysis of rbES cells produced using 3C versus iFLY media revealed widespread differential gene expression. In the feeder-free 3C system, core pluripotency genes (*OCT4*, *SOX2*, and *NANOG*) and partial naïve/formative state markers (*KLF17*, *ZFP42*, *LIN28B,* and *OTX2*) were significantly upregulated, whereas primed state markers (*NODAL* and *TBXT*) were dramatically downregulated (Figure 4). IWP2 inhibits the Wnt palmitoyltransferase Porcupine, thereby blocking Wnt protein palmitoylation and secretion, and downregulating the Wnt/β-catenin pathway (Mo et al., 2013). IWP2 supports the self-renewal of rabbit embryonic stem cells and inhibits Wnt-induced mesodermal and endodermal differentiation (Kobayashi et al., 2021). Earlier work has demonstrated that naïve mouse ES cells sustain naïve transcriptional programs and retain chimeric competence in the absence of Wnt signaling (Lyashenko et al., 2011; Biechele et al., 2013). We supplemented the culture medium with IWP2, a small-molecule inhibitor of the Wnt acyltransferase Porcupine that blocks Wnt protein secretion and thereby inhibits canonical Wnt/β-catenin signaling (Kobayashi et al., 2021). Accordingly, Wnt-related ligand expression was markedly reduced in the 3C culture system, probably owing to the excessive intracellular accumulation of these ligands in the endoplasmic reticulum and consequent impairment of their secretion. The addition of IWP2 markedly increased proliferation and colony formation, whereas its withdrawal precipitated rapid differentiation of rbES cells.

TSA is an HDAC inhibitor that promotes histone acetylation, opens the chromatin structure, and increases gene expression (Lee et al., 2025). Transcriptional reprogramming mediated by histone deacetylase inhibition is tightly correlated with rapid dynamic alterations in the activating H3K4me3 and repressive H3K27me3 modifications of histone H3. Our results revealed that high concentrations of TSA significantly increased the enrichment levels of H3K4me3, H3K27me3 and H3K27ac in rabbit embryonic stem cells (rbESCs), while low concentrations of TSA effectively maintained the pluripotent characteristics of rbESCs. The underlying mechanism may be that TSA regulates the epigenetic modification homeostasis of rbESCs in a dose-dependent manner. Low-dose TSA moderately inhibits HDAC activity, stabilizes the balanced deposition of H3K4me3, H3K27me3 and H3K27ac, and consequently sustains the pluripotency and self-renewal capacity of stem cells (Farahi et al., 2022). By contrast, high-dose TSA strongly suppresses HDAC function, markedly elevates global levels of H3K4me3, H3K27me3 and H3K27ac, remodels chromatin configuration, and ultimately disrupts cellular pluripotency homeostasis (Karantzali et al., 2008). DZNep is a small molecule that is an indirect inhibitor of the histone methyltransferase EZH2, an enzymatic subunit of polycomb repressive complex 2 (PRC2) (Zhang et al., 2025). DZNep induces the depletion of EZH2 protein and reduces the H3K27me3 modification level that may be elevated by TSA, thereby promoting a more open chromatin state and altering the expression patterns of target genes (Girard et al., 2014). DZNep enhances the efficiency of establishing pluripotency networks by loosening repressive chromatin, making it easier for ES cells to acquire and maintain pluripotency (Cao et al., 2023). However, prolonged DZNep exposure may also destabilize pluripotency once the fate of stem cells is reprogrammed (Zhao et al., 2018). Therefore, we hypothesized that combined treatment of ES cells with IWP2+DZNep + TSA can create a highly open chromatin state (TSA + DZNep), remove polycomb repression of pluripotency genes, especially for naïve-state genes, and block the Wnt signaling pathway (IWP2) which otherwise promotes mesoderm/endoderm cell determination and cell fates. As previously reported by Liu et al., the 4CL culture system containing PD, IWR1, DZNep and TSA enables efficient conversion of monkey embryonic stem cells from primed to naïve state, while sustaining balanced genome-wide DNA demethylation, elevated naïve pluripotency gene expression and well-preserved genomic stability (Cao et al., 2023). Moreover, growing evidence indicates that the mechanical state of cells is one of the key factors determining cell identity (Lu et al., 2025). We observed that the nuclei of cells cultured in iFLY medium were significantly larger than those under 3C conditions: cells in the 3C system showed ordered proliferation, stable pluripotency homeostasis and tight chromatin packaging, while iFLY-cultured cells had higher heterogeneity, with some in a transcriptionally active state and relaxed chromatin, resulting in larger nuclei. Reversion from primed to naïve pluripotency is a complex process that requires extensive reprogramming of gene expression and signaling networks. For example, ectopic expression of *NANOG* or *KLF2* converts human pluripotent stem cells (PSCs) into ground-state ES cells (Takashim et al., 2014). In human cells, such resetting elicits global changes in DNA methylation and the transcriptome that are consistent with a more primitive state (Takashim et al., 2014). In human PSCs, combined treatment with the HDAC inhibitor TSA and the PRC2 inhibitor DZNep synergistically activated the 8C-like cell core circuitry, including *ZSCAN4*, *DUXA*, *ZNF280A*, *TPRX1*, and *DPPA3,* driving cells from pluripotency toward an eight-cell embryo-like totipotent state (Mazid et al., 2022). Use of the same dual inhibition in our rbES system further elevated pluripotency, promoted compact “nest-like” colonies, and sharply reduced differentiation. Notably, RNA-seq revealed upregulation of HDAC1 and DNMT3B under 3C culture conditions with TSA and DZNep treatment, which likely arises from a compensatory transcriptional response triggered by the inhibitors to restore epigenetic homeostasis, resulting in the seemingly paradoxical observation of increased gene expression despite suppressed enzymatic activity. Conversely, the Wnt agonist CHIR99021 or the MEK inhibitor PD0325901 compromised rbES cell identity and pluripotency in iFLY-derived rbES cells. Similarly, a high dose of CHIR99021 significantly repressed *POU5F1*, whereas even a low dose of PD0325901 abolished pluripotency, mirroring observations in bovine ES cells (Zhao et al., 2021). mES cells rely on Wnt pathway activation to maintain naïve pluripotency. In the classical 2i culture system, CHIR99021 activates Wnt/β-catenin signaling by inhibiting GSK3β, which stabilizes the ground-state characteristics and suppresses spontaneous differentiation, representing a canonical strategy for the culture of mouse ES cells. In contrast, ES cells from large animals such as humans and rabbits exhibit distinct species-specific regulatory patterns (Du and Wu, 2024). Excessive Wnt activation disrupts pluripotency homeostasis and triggers lineage specification and differentiation. Conversely, moderate Wnt inhibition facilitates the stabilization and maintenance of the naïve pluripotent state and restricts differentiation propensity. Consistently, most reported culture media for human naïve/formative ES cells contain Wnt pathway inhibitors (Kobayashi et al., 2021; Bredenkamp et al., 2019; Khan et al., 2021; Bayerl et al., 2021). The regulatory effect of CHIR99021 on rabbit ES cell pluripotency displays a prominent dose dependence. High concentrations of CHIR99021 markedly downregulate the core pluripotency gene POU5F1 and impair cell identity and colony morphology. These findings indicate that the basal Wnt signaling level must be tightly controlled in rabbit ES cells; even mild Wnt activation can perturb the balance of epigenetic and pluripotency regulatory networks and drive cell differentiation. This differs substantially from mouse ES cells, which tolerate and depend on CHIR99021-induced Wnt activation. Accordingly, supplementation with Wnt inhibitors such as IWP2, rather than Wnt activation by CHIR99021, is more appropriate for maintaining pluripotency stability in rabbit ES cell culture systems. Collectively, these data demonstrate that Wnt/β-catenin inhibition combined with Raf/MEK/ERK activation is essential for stabilizing pluripotency in rbES cells.

Bidirectional interconversion between the naïve and primed pluripotent states has been achieved in both mice and humans (Shahbazi et al., 2017). An epiblast-like cell (EpiLC) intermediate state, termed formative state, has also been captured (Du and Wu, 2024). The formative state functions as a developmental bridge between the naïve and primed pluripotent phases, endowing cells with the competence for multi-lineage commitment. The formative pluripotency markers *LIN28B* and *OTX2* were expressed, while the primed marker TBXT was nearly absent in the 3C feeder-free system (Figure 2; Supplementary Figures S2). Mouse *Otx2* is a canonical marker of this formative identity (Smith, 2017; Morgani et al., 2017). In naïve mouse ESCs and primed epiblast stem cells (EpiSCs), *Otx2* is dispensable (Acampora et al., 2013); its knockout or *Nanog* overexpression can even preserve the naïve state under LIF + FBS treatment because *Otx2* directly occupies the *Nanog* promoter to modulate its transcription and thereby influences mES cell heterogeneity (Acampora et al., 2016; Acampora et al., 2017). However, once mES cells transition toward the formative state, Otx2 null cells cannot be stably maintained and instead undergo sustained neural differentiation (Kinoshita et al., 2021). Intriguingly, rbES cells derived from the 3C system expressed high levels of OTX2, whereas the transcript was absent in primed iFLY rbES cells. This observation implied that some 3C rbES cells were at the transition between the naïve and primed state. Moreover, we found that other naïve pluripotency genes, including *KLF4*, *ESRRB*, and *TBX3*, were downregulated in 3C-cultured rbES cells. These genes serve as naïve-specific markers and are highly expressed only in the naïve pluripotent state (Bouchereau et al., 2022). We propose that *KLF4* and *ESRRB* are canonical downstream targets of the Wnt/β-catenin pathway, whose transcription is directly activated by Wnt signaling (Fan et al., 2020; Ai et al., 2016). In the 3C system, IWP2 blocks Wnt ligand secretion and inhibits canonical Wnt activity, reducing nuclear β-catenin accumulation and attenuating its transcriptional induction of *KLF4* and *ESRRB*, consequently resulting in their downregulated expression. Transcriptomic heatmap analysis reveals the unique advantages and precise regulatory capacity of the 3C system in governing rbES pluripotency. It not only suppresses lineage differentiation of primed cells cultured in the iFLY system, but also crucially drives cells into a formative pluripotent state bridging the naïve and primed phases. When primed rbESCs are cultured in 3C medium, microenvironmental signals derived from small-molecule inhibitors and Matrix substrate efficiently reprogram cell fate from the primed toward the naïve pluripotent state. During this process, canonical primed lineage genes (e.g., *FOXA1*, *LEF1*, *SOX1*) are significantly downregulated, which maintains pluripotency and prevents premature lineage specification, and further improves the controllability of cell differentiation potential. Meanwhile, key naïve pluripotency markers (e.g., *ZFP42*, *KLF17*) are upregulated, indicating that the 3C system confers partial epigenetic reprogramming ability to push cells toward a more primitive pluripotent state. We hypothesize that long-accumulated epigenetic memory in primed cells, including aberrant DNA methylation and disordered histone modifications, can only be thoroughly eliminated through systematic epigenetic remodeling mediated by specific signaling pathways. Limited inductive strength of the 3C system cannot overcome intrinsic epigenetic barriers, consequently resulting in incomplete transcriptional activation of core naïve pluripotency genes (Nishino et al., 2011; Brix et al., 2015). Collectively, these findings indicate that rbES maintained in the 3C system do not fully recapitulate the naïve pluripotent state of the ICM; instead, they occupy an intermediate state between the naïve pluripotency of the ICM and the primed state of cells cultured in the iFLY system, retaining the differentiation-suppressive features of naïve cells while acquiring partial transcriptomic characteristics of the primed state. This is consistent with the phenotypic observation in the present study that the cells displayed a nest-like morphology rather than the characteristic dome-shaped morphology of naïve pluripotent stem cells. However, the precise molecular mechanisms underlying this poised state remain to be elucidated in depth.

PGC differentiation is one of the most fascinating reprogramming mechanisms and requires a unique combination of signaling pathways and gene regulatory rewiring, which separates PGCs from somatic cell fates (Yu et al., 2025). In this study, we successfully differentiated PGCLCs, after induction of 3C-derived rbES cells using BMP4, EGF, and SCF, with highly expressed PGC-specific genes including *PRDM1* (epigenetic reprogramming) (Zhou et al., 2024), *TFAP2C* (germline gene activation) (Tang et al., 2022), *NANOS3* (germ cell survival, migration and maintenance) (Wang et al., 2025) and *SOX17* (upstream determinant of PGC fate) (Yu et al., 2025), respectively (Figure 6). In mice, PGCs arise after implantation from posterior epiblast cells exposed to BMP/Wnt, which activate a transcriptional-epigenetic program with expression of key factors including *BLIMP1*, *PRDM14*, and *TFAP2C*, which reprogram PGCs into the germline lineage, followed by migration to the gonads (Magnúsdóttir et al., 2013). The transition of mES cells into EpiLCs *in vitro* opens the PGC competent reprogramming window (Ohinata et al., 2009; Hayashi et al., 2011; Wang et al., 2021). Within naïve mES cells, the *Prdm14* promoter displays bivalent chromatin (H3K4me3/H3K27me3), and PRDM14 itself restrains DNA methyltransferases, thereby preserving epigenetic plasticity (Kinoshita et al., 2021; Bernstein et al., 2006). In contrast, primed mouse EpiSCs lose these markers, silence *Prdm14*, and lose the ability to form PGCLCs (Hayashi et al., 2011). In humans, PGCs express specific genes, including *SOX17*, *BLIMP1*, *TFAP2C,* and supportive *PRDM14* (Irie et al., 2015). In rabbit *in vivo* embryos, PGCs first emerge in the posterior epiblast at the pre-streak stage and subsequently migrate into the mesoderm (Kobayashi et al., 2021). This route differs from that in mice and more closely resembles human development (Yamashiro et al., 2020). TFAP2C functions as a core determinant of germ cell fate, directly activating germline-specific genes such as *Prdm1* during mES cell to PGC specification (Ohinata et al., 2005; Nakaki et al., 2013). Toshihiro et al. identified BMP4 as the pivotal morphogen for PGC fate during rbES cell specification and demonstrated that Wnt inhibition blocks PGCLC emergence (Kobayashi et al., 2021). Functional PGCLCs were efficiently generated through precise modulation of the Wnt and BMP signaling pathways, and SOX17, rather than BLIMP1 as in mice, was identified as the key transcription factor in rabbits (Kobayashi et al., 2021). Recent evidence further reveals that OTX2 physically associates with TFAP2C and co-occupies target loci to cooperatively drive human PGCLC induction (Zhang et al., 2025). During induction, we lowered the BMP4 concentrations and supplemented the cells with CHIR99021 and retinoic acid. Under these conditions, rbES cells retained exquisite responsiveness to BMP4 and efficiently generated PGCLCs. qPCR analysis of PGCLCs differentiated from 3C-derived rbES cells at day 3.5 revealed elevated expression of canonical early germ cell markers including *NANOS3*, *TFAP2C*, *PRDM1* and *SOX17*. Positive VASA expression was further detected on day 10 of induction, demonstrating that these rbES cells are fully competent for germline differentiation and capable of maturing into germ cells.

In summary, the transition of rbES cells from the primed (iFLY) to the formative state was achieved by extensive molecular reprogramming using three small-molecule compounds (IWP2+DZNep + TSA), defined as the feeder-free 3C culture system. rbES cells derived from the 3C system expressed a subset of both naïve and formative pluripotency genes, maintained robust pluripotency and long-term self-renewal, while retaining the potential for germline differentiation into PGCLCs.

## 5 Conclusion

This study demonstrated that the feeder-free 3C culture system (IWP2+DZNep + TSA) captures an formative state during the transition of rbES from the primed to the naïve state, which entailed global transcriptional reprogramming to reset core and naïve pluripotency and reshaped regulatory gene expression and self-renewal. The 3C system not only maintains stable pluripotency and self-renewal but also poises cells for germline differentiation; upon subsequent exposure to BMP4, SCF, and EGF, it enables efficient induction of PGCLCs within 3.5 days. This study still has certain limitations: due to current technical and platform constraints, the germline chimera assay, the gold standard for authentic naïve pluripotency identification, has not been performed. We will prioritize establishing embryo chimera models and evaluating germline contribution in our future research. This 3C platform for ES-cell differentiation provides a faithful and efficient system for dissecting the molecular and epigenetic circuits that govern rbES cell plasticity toward either pluripotency or directed germ-cell induction.

## Data Availability

The datasets presented in this study can be found in online repositories. The names of the repository/repositories and accession number(s) can be found below: https://ngdc.cncb.ac.cn/gsa/browse/CRA026197, GSA: CRA026197.
