# Supplementary figures and images for: Novel feeder-cell-free 3C system promotes the transition from primed to formative pluripotency, self-renewal, and germline differentiation in rabbit embryonic stem cells in vitro
Source: Front Cell Dev Biol. 2026 Jun 8;14:1841436. doi: 10.3389/fcell.2026.1841436 (PMC13283986; doi:10.3389/fcell.2026.1841436)

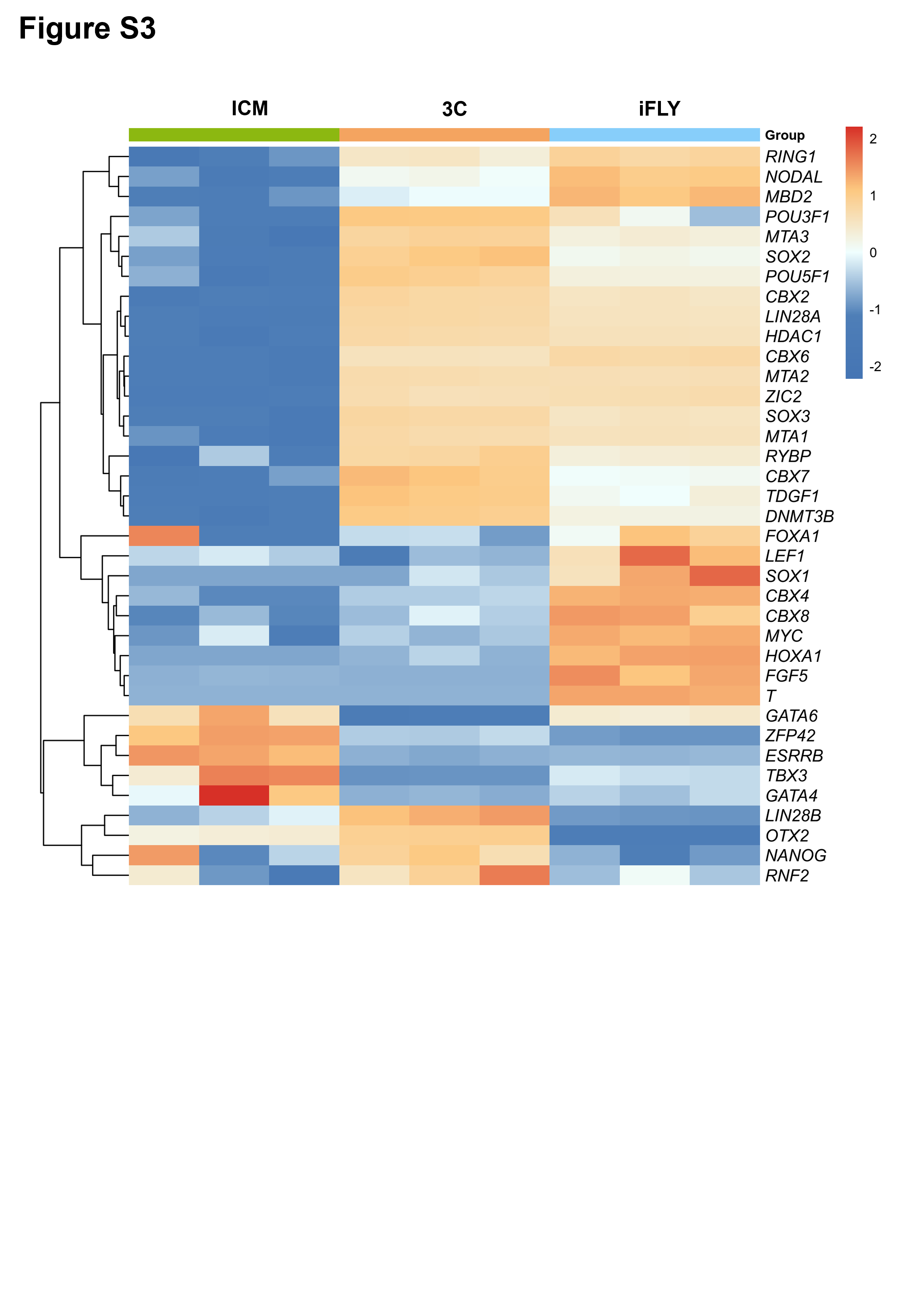

Supplement: Supplementary file 1 [file Image3.tif]

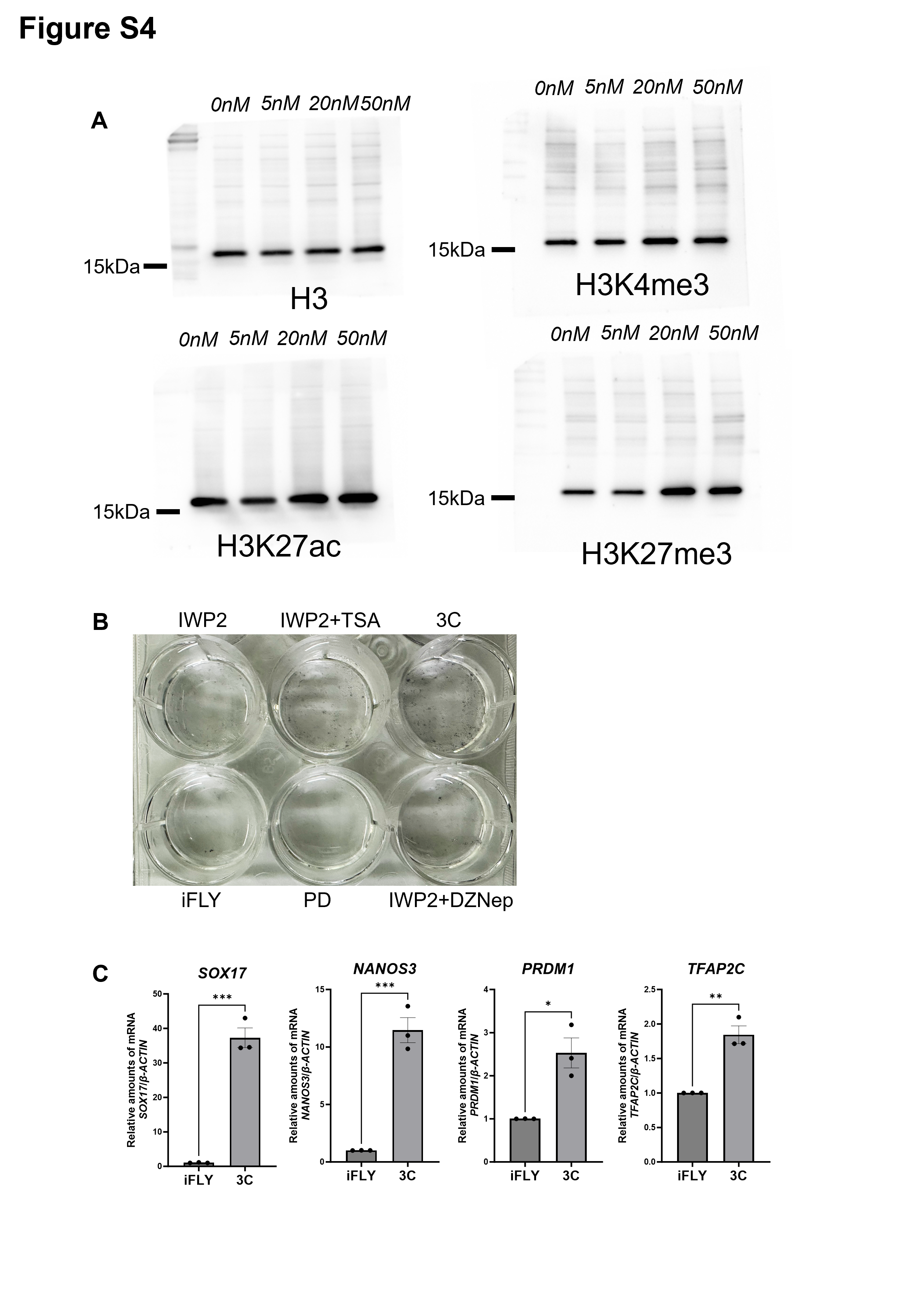

Supplement: Supplementary file 2 [file Image4.tif]

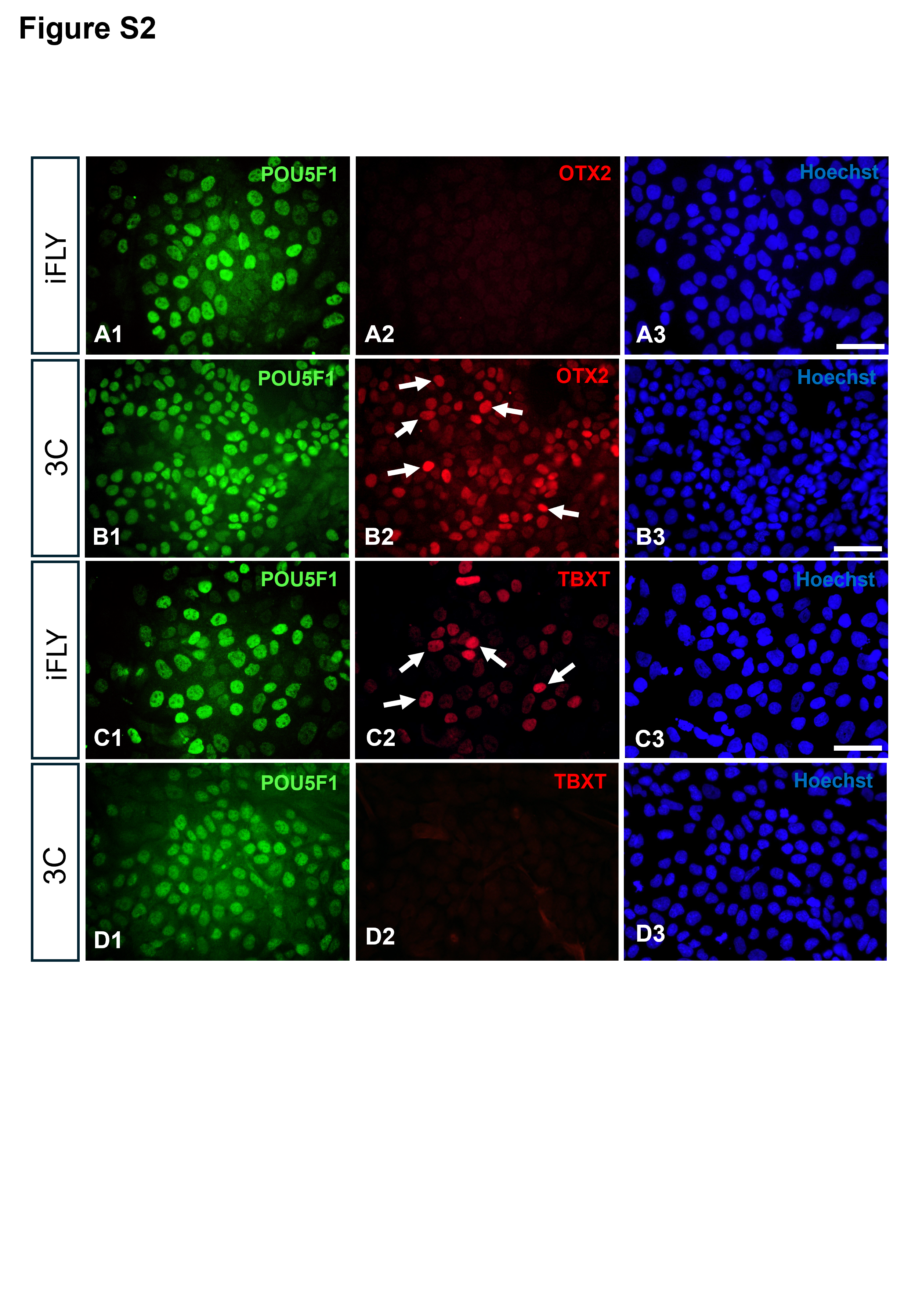

Supplement: Supplementary file 3 [file Image2.tif]

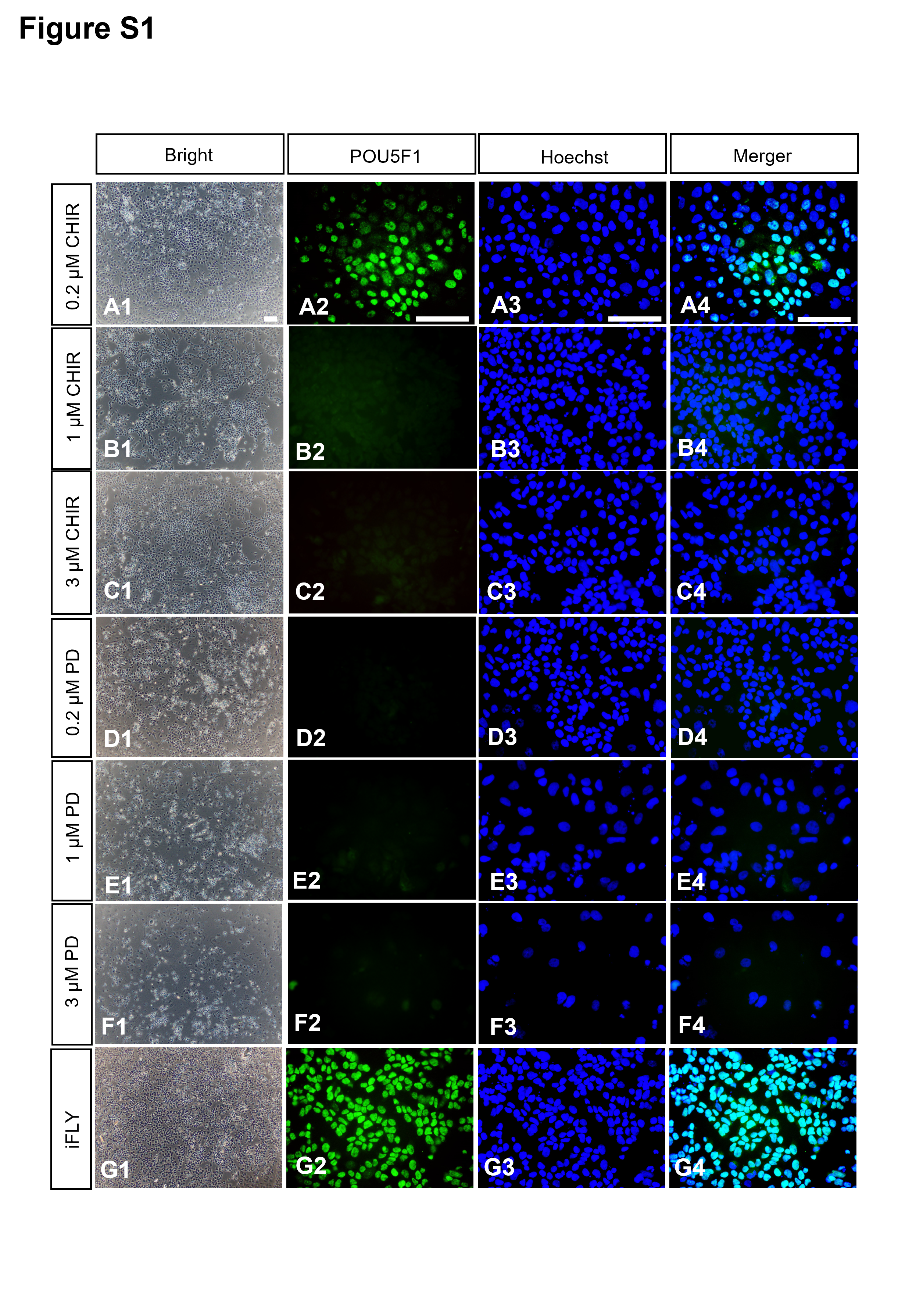

Supplement: Supplementary file 4 [file Image1.tif]
